# Aridity thresholds of soil microbial metabolic indices along a 3,200 km transect across arid and semi-arid regions in Northern China

**DOI:** 10.7717/peerj.6712

**Published:** 2019-04-09

**Authors:** Jianfeng Hou, Feike A. Dijkstra, Xiuwei Zhang, Chao Wang, Xiaotao Lü, Peng Wang, Xingguo Han, Weixin Cheng

**Affiliations:** 1 Erguna Forest-Steppe Ecotone Research Station, Institute of Applied Ecology, Chinese Academy of Sciences, Shenyang, Liaoning, China; 2 University of Chinese Academy of Sciences, Beijing, Beijing, China; 3 Sydney Institute of Agriculture, School of Life and Environmental Sciences, University of Sydney, Sydney, Camden, Australia; 4 Institute of Wetland Ecology & Clone Ecology; Zhejiang Provincial Key Laboratory of Evolutionary Ecology and Conservation, Taizhou University, Taizhou, Zhejiang, China; 5 Institute of Botany, Chinese Academy of Sciences, Beijing, Beijing, China; 6 Environmental Studies Department, University of California, Santa Cruz, Santa Cruz, CA, USA

**Keywords:** Respiration, Microbial metabolism, Metabolic quotient, Carbon limitation, Microbial biomass, Microbial biomass.

## Abstract

Soil microbial processes are crucial for understanding the ecological functions of arid and semi-arid lands which occupy approximately 40% of the global terrestrial ecosystems. However, how soil microbial metabolic activities may change across a wide aridity gradient in drylands remains unclear. Here, we investigated three soil microbial metabolic indices (soil organic carbon (SOC)-based microbial respiration, metabolic quotient, and microbial biomass as a proportion of total SOC) and the degree of carbon limitation for microbial respiration along a 3,200 km transect with a wide aridity gradient. The aridity gradient was customarily expressed using the aridity index (AI) which was calculated as the ratio of mean annual precipitation to mean annual evaporation, therefore, a lower AI value indicated a higher degree of aridity. Our results showed non-linear relationships between AI values and the metabolic indices with a clear aridity threshold for each of the three metabolic indices along the aridity gradient, respectively (AI = 0.13 for basal respiration, AI = 0.17 for metabolic quotient, and AI = 0.17 for MBC:SOC ratio). These metabolic indices linearly declined when AI was above the thresholds, but did not show any clear patterns when AI was below the thresholds. We also found that soil microbial respiration was highly limited by available carbon substrates at locations with higher primary production and relatively lower level of water limitation when AI was above the threshold, a counter-intuitive pattern that microbes were more starved in ecosystems with more substrate input. However, the increasing level of carbon limitation did correspond to the declining trend of the three metabolic indices along the AI gradient, which indicates that the carbon limitation influences microbial metabolism. We also found that the ratio of microbial biomass carbon to SOC in arid regions (AI < 0.2) with extremely low precipitation and primary production were not quantitatively related to SOC content. Overall, our results imply that microbial metabolism is distinctively different in arid lands than in non-arid lands.

## Introduction

Arid and semi-arid lands cover 41% of the global land area, supporting the livelihoods of more than two billion people on Earth. These drylands are also vulnerable to climate change and other disturbances (Adeel, 2004; Reynolds et al., 2007). Scientific understanding of these vulnerabilities is required for maintaining and improving the ecosystem services and sustainability of these drylands. Soil microbial processes, such as heterotrophic respiration and metabolic activities, are fundamental to ecosystem functions. Particularly, understanding these soil microbial processes are critical for predicting changes in the terrestrial ecosystems in response to global climate change (Schimel & Schaeffer, 2012; Tian et al., 2014). Because inadequate water supply is a key feature of these drylands, investigating patterns of change in soil microbial processes across an aridity gradient can be informative for better understanding of these drylands (Yao et al., 2017; Jones et al., 2018).

Soil heterotrophic microbial respiration (commonly measured as the rate of CO_2_ production in root-free soil) is a key component of soil microbial processes. Based on a recent study using data from mostly non-arid sites (Colman & Schimel, 2013), soil microbial respiration is primarily influenced by a combination of climatic variables (i.e., temperature and precipitation), soil organic carbon (SOC) concentration, microbial biomass, and clay content, of which microbial biomass alone is responsible for approximately 60% of the variation in soil microbial respiration. Other studies have also shown that soil microbial respiration can be influenced by a number of biotic and abiotic factors in drylands (Schlesinger & Pilmanis, 1998; Collins et al., 2014; Ahlstrom et al., 2015; Chandregowda, Murthy & Bagchi, 2018). However, how soil microbial respiration changes across a wide aridity gradient in drylands remains unclear and warrants more studies.

Soil microbial metabolic activities normally increase in response to addition of readily available carbon substrates, such as glucose (Anderson & Domsch, 1978). The level of microbial respiratory response to glucose addition has been considered as an index of microbial carbon limitation (Parkinson & Coleman, 1991; Cheng et al., 1996, Gershenson, Bader & Cheng, 2009; Tian et al., 2014). The carbon-limitation of soil microbial activity has been demonstrated by the 2–6-fold increases in soil respiration immediately after the addition of a readily available substrate (glucose is the most commonly used one) at or above the saturation level (Anderson & Domsch, 1978; Pang et al., 2015). The microbial respiration after addition of available substrate at the saturation level has been referred as the substrate-induced respiration (SIR), which remains nearly constant during the initial 4 h period before any microbial growth is noted (Lin & Brookes, 1999; Mingorance & Pena, 2016). Logically, the higher the ratio of SIR rate to the basal respiration rate (respiration rate without substrate addition), the higher the level of carbon limitation. Therefore, we define this ratio as soil microbial carbon limitation index (CLI). How soil microbial CLI will change across a wide aridity gradient in drylands remains an open question.

Another microbial index is the metabolic quotient (qCO_2_), defined as the basal respiration rate per unit of microbial biomass (Anderson & Domsch, 1985). Because SIR is often linearly and positively correlated to microbial biomass (Anderson & Domsch, 1978), qCO_2_ should be inversely related to CLI. The qCO_2_ has been used as a crucial parameter in recent models of soil carbon cycling (Allison, Wallenstein & Bradford, 2010; Wang, Post & Mayes, 2013; Wieder, Bonan & Allison, 2013; Sulman et al., 2014), and has been widely used in assessing soil microbial metabolic status (Wardle & Ghani, 2018). A recent synthesis based on data gathered from 210 published papers demonstrated large variability of the qCO_2_ across different biomes, especially between croplands and natural ecosystems (Xu et al., 2017). Furthermore, this synthesis also showed that the qCO_2_ tends to increase with increased temperature, in accordance with an earlier study (Insam, 1990). To what extent the qCO_2_ may vary with aridity has been inadequately addressed. This inadequacy clearly requires more attention, considering that better understanding of water regulation on soil microbial processes are highly needed for improving global carbon models and land use strategies (Reynolds et al., 2007; Luo et al., 2017).

Overall soil microbial metabolic activity and microbial biomass carbon (MBC) are ultimately connected to SOC because SOC is the primary substrate source supporting microbial biomass (Insam & Domsch, 1988; Insam, Parkinson & Domsch, 1989; Insam, 1990). The MBC:SOC ratio has been used as an indicator of microbial substrate assimilation and metabolism (Xu et al., 2014). The ratio varies with climate conditions (i.e., temperature, precipitation, and evapotranspiration) (Insam, Parkinson & Domsch, 1989; Insam, 1990) and with the level of ecosystem disturbances (Insam & Domsch, 1988). Some studies have shown that the MBC:SOC ratio tends to be higher in relatively dry ecosystems (Insam, Parkinson & Domsch, 1989; Xu et al., 2014). However, how this ratio may vary across a wide aridity gradient has not been adequately investigated.

In this study, we aimed to explore the patterns and thresholds in the relationship between soil microbial metabolism and climatic aridity by using soil samples taken from a 3,200 km transect, which covers a wide aridity gradient across arid and semi-arid regions in Northern China. Microbial respiration, qCO_2_, and the MBC:SOC ratio were our three main microbial metabolic indices, which were measured and used to assess the overall microbial metabolism. To assess these three metabolic indices and microbial carbon limitation, we also calculated soil microbial CLI. We quantified the aridity gradient by calculating the Aridity Index (United Nations Environment Programme (UNEP), 1992) (AI: the ratio of annual precipitation to annual potential evapotranspiration (PET)) using long-term meteorological data. The AI as defined in this way has an inverse relationship with the actual aridity; that is, a lower AI value indicates more dryness. We further explored how these patterns and thresholds may relate to aboveground net primary production and other soil properties.

## Materials and Methods

### Study areas

**Figure 1:**
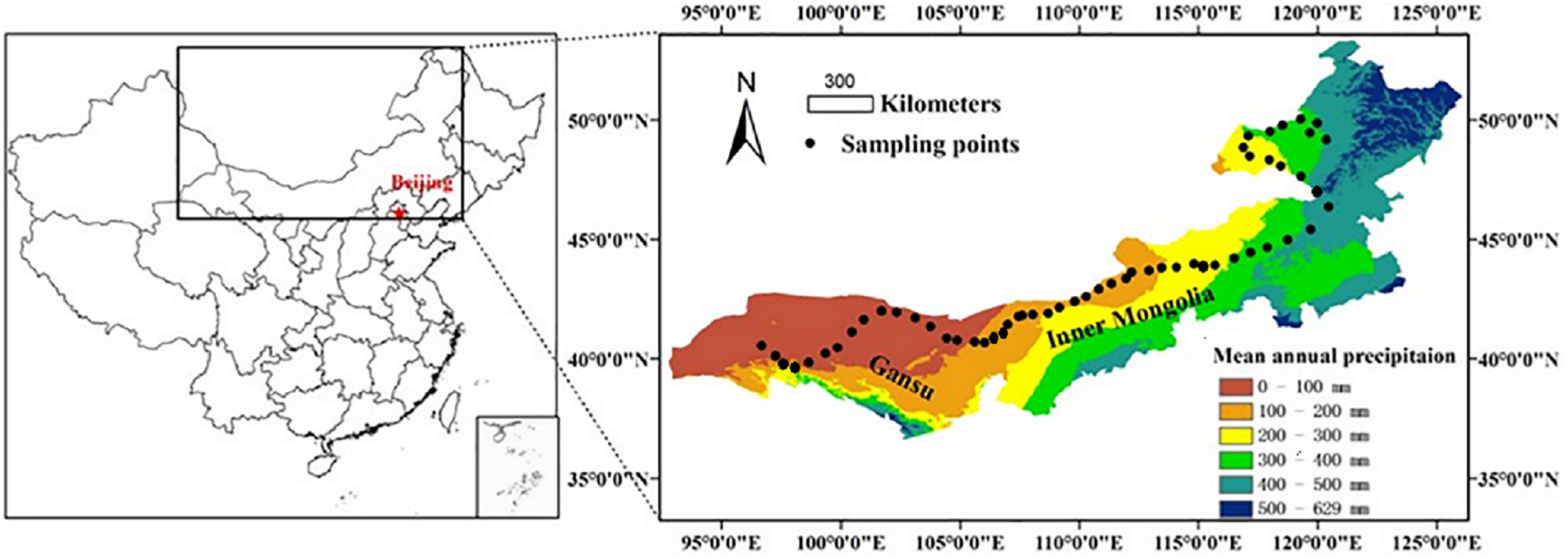
A sampling transect of 3,200 km across arid and semi-arid regions in Northern China. A sampling transect of 3,200 km across arid and semi-arid regions in Northern China. A total of 56 sampling sites were selected (black dots) along this transect. Scale bar is 300 km.

This study was conducted along a 3,200 km west-east transect of arid and semi-arid regions in northern China, which spans three regions (Xinjiang, Gansu, and Inner Mongolia) (Fig. 1). The longitude of this transect ranged from 96°40′ to 120°28′E, and the latitude from 40°07′ to 50°1′N. Mean annual precipitation (MAP) ranged from 34 to 436 mm and mean annual air temperature (MAT) ranged from −5 to 10 °C. Annual PET declined from about 1,200 mm in the west to about 710 mm in the east. MAT was negatively correlated with MAP along the transect (*R*^2^ = 0.889, *P* < 0.001, Fig. S1 in the supporting information). By including both MAP and MAT, the AI provides an integrated index of variation of climate (Eq. (1)) (Arora, 2002; Delgado-Baquerizo et al., 2013; Luo et al., 2015). The AI of this transect ranged from 0.03 to 0.56 and was calculated using the following equation:

(1) $${\rm AI} = \displaystyle{{{\rm MAP}} \over {{\rm PET}}}$$

The main vegetation types were desert steppe in the western part, typical steppe in the middle, and meadow steppe in the eastern part of the transect. The aboveground net primary productivity (ANPP) ranged from 0 (no plants) in the desert steppe to >400 g m^−2^ year^−1^ in the meadow steppe. Soil types were predominantly arid, sandy, brown loess rich in calcium, which belonged to the order of Aridisols (for the dryer side of the transect) and the order of Mollisols (for the relatively moist side of the transect) in the USDA soil classification system.

### Soil sampling

Sampling over the entire transect was conducted in July–August 2012. A total of 56 sampling sites were chosen, they were all under natural conditions without any significant human disturbance. All longitudinal, latitudinal, and altitudinal data were obtained by using a GPS system (eTrex Venture, Garmin, ±3 m accuracy). At each sampling site, two large plots (50 × 50 m), 1.0 km apart from each other, were setup first, and then five subplots (1 × 1 m each) (or five 5 × 5 m subplots in are as dominated by shrubs) were further located within each large plot randomly. In each subplot, all the aboveground plants were harvested, sorted by different species, and stored for determining ANPP. After harvesting, five random soil samples were collected with a soil core (0–10 cm deep, 2.5 cm diameter) and then bulked as one composite sample for further analysis. In this way, we collected 10 replicated plant and soil samples at each site. We used these 10 soil samples at each site as analytical replicates and the mean of the 10 samples as representing the site for all subsequent measurements. A total of 560 soil samples were collected along the entire transect, but a smaller number of plant samples was collected because there were sites without any vegetation. The distance between two adjacent sites was kept at 50–100 km. All soil samples were brought to the laboratory and then air dried, passed through a 2 mm sieve, and homogenized by hand mixing. Visible organic debris was removed from each soil sample by hand-picking. All soil samples were stored at 20 °C after air-drying until further analyses. Results from a relevant study (Zornoza et al., 2007) indicated that air-drying soil samples from arid and semi-arid ecosystems is appropriate for measuring MBC, basal respiration, metabolic quotient, and water soluble carbon.

### Climate data

The MAT (°C) and MAP (mm) data (1950–2000) were used for calculating AI values at each sampling site, and were extracted from a global climate dataset with a resolution of 0.0083 × 0.0083° latitude by longitude (approximately one km^2^ at the equator, obtained from WorldClim: http://www.worldclim.org/). The PET data (1950–2000) were extracted from the CGIAR-CSI Global AI and Global Potential Evapo-Transpiration Climate Database (http://www.cgiar-csi.org/data/global-aridity-and-pet-database).

### Measuring soil basal respiration and SIR

For each site (Fig. 1), 10 field replicates for each soil sampling sites were pre-incubated for 21 days at 25 °C inside processor-controlled incubators (SHELLAB LI20-2; Sheldon Manufacturing Inc., Cornelius, OR, USA, with a temperature control accuracy and evenness of ±0.02 °C) before soil respiration measurements. Briefly, 100 g dry weight soil was placed in a polypropylene random (PPR) plastic container (30 cm in height, and 2 cm in diameter). Both ends of the PPR containers were closed by one-hole silicone rubber stoppers. A piece of flexible plastic tubing was connected to each rubber stopper. The soil moisture inside each container during incubation was carefully maintained at 60% water hold capacity (WHC) by weighing regularly and adding with deionized water during the preincubation time. Continuous aerobic condition in each pipe was maintained by an automatic timer-controlled aeration system which aerated each pipe with room air at a rate of 80 mL min^−1^ for 1 h at 4-h intervals.

After the pre-incubation, soil basal respiration was immediately measured directly in the soil columns placed in the PPR plastic container by a soil respiration system originally described by Cheng & Virginia (1993) and further modified by Zhang et al. (2017).This system consists of a LICOR CO_2_ analyzer (Li-6262; Li-COR Biosciences, Lincoln, NE, USA) a water bath for maintaining a stable temperature at 25 °C, a mass flow meter (GFM; Aalborg Ltd, Orangeburg, New York, USA) for determining air flow velocity, and an air pump connected with a soda-lime column which provided CO_2_-free air. The CO_2_-free air was passed through a copper coil submerged in the water bath for equilibrating the air temperature with the water bath temperature before reaching a manifold with 30 outflow tubes. Each outflow tube had an inline fine metering needle valve for stabilizing the airflow rate at 60 mL min^−1^ before the CO_2_-free air passed through the plastic pipe with pre-incubated soil. One of the outflow tubes was connected to an empty plastic container and was used as a blank reference. After a 1-h stabilizing period in the water bath, each individual air out flow tube from each plastic pipe was connected in turn to a Li-COR 6262 infrared gas analyzer with a mass flow meter, and the CO_2_ concentration and air flow rate were recorded after approximately 5 min. The respiration rate for each sample was calculated using the CO_2_ concentration, the air flow rate and the exact amount of soil in each pipe.

After measuring the soil basal respiration, glucose solution (60 g L^−1^ concentration) was added to each PPR pipe using a syringe with a long needle to bring the soil water content to 100% of the WHC. After a 2-h stabilizing period, the SIR was measured using the same procedure as used for basal respiration described above. The amount of added glucose to each sample ranged from 6 to10 mg glucose g^−1^ soil, which was at or above the saturation level for a maximum microbial respiratory response (Cheng & Coleman, 1989). All measurements were conducted within 1 year after the initial soil sampling time. After measurements for soil respiration, 20 g soil was taken out from the PPR tubes for determining the dry weight and the water content of each soil sample.

### Analysis of soil properties

Microbial biomass carbon was determined by using the chloroform fumigation-extraction method after pre-incubation (Joergensen, 1996). Briefly, 20 g of pre-incubated fresh soil was fumigated with pure chloroform for 24 h, then the chloroform was completely removed from soil samples by repeated vacuuming. Both fumigated and unfumigated subsamples from each soil sample were extracted with 40 mL 0.5M K_2_SO_4_ solution after shaking for 1 h. The total organic carbon in the extract of each sample was analyzed using a multi N/C^®^ 3100 total organic carbon analyzer (Analytik Jena, Jena, Germany). The MBC was calculated by using the difference in extracted organic carbon between fumigated and unfumigated samples and divided by an efficient factor (K_EC_) of 0.45. SOC and total nitrogen contents were determined using an elemental analyzer (Model CN; Elementar Analysen Systeme GmbH, Langenselbold, Germany).

### Calculations

The basal respiration rate and the SIR rate (respiration rate after glucose addition) of each soil sample was calculated using the following equation from (Gershenson, Bader & Cheng, 2009):

(2) $${R_s} = \displaystyle{{0.536 \times ({C_c} \times {R_f}{\rm )}} \over {{W_s}}}$$

Where *R_s_* is the soil respiration rate as measured by the soil CO_2_ efflux rate (µg CO_2_-C g^−1^ dry soil h^−1^), *C_c_* is the recorded CO_2_ concentration (µmol CO_2_ mol^−1^), *R_f_* is the recorded flow rate (mL h^−1^), and *W_s_* is the dry weight (g) of the sample.

Using both the basal respiration rate and the SIR for each soil sample, we calculated the CLI for the soil sample using the following equation:

(3) $${\rm CLI} = \displaystyle{{{R_{{\rm Glu} + }}} \over {{R_{{\rm Glu} - }}}}$$

where *R*_Glu+_ and *R*_Glu−_ are the respiration rates at 25 °C with and without added-glucose, respectively (Cheng et al., 1996).

The qCO_2_ for each soil sample was calculated as the basal respiration rate per unit of MBC (Anderson & Domsch, 1993):

(4) $${\rm{qC}}{{\rm{O}}_2} = {{{{R_{{\rm{Glu}}}}_ - }}\over{{{\rm{MBC}}}}}$$

where qCO_2_ is the metabolic quotient, *R*_Glu−_ is the respiration rate at 25 °C without glucose addition.

### Data analysis

To explore potential patterns and thresholds, we employed OriginLab 8.5 and R version 3.02 (R Core Team, 2013) for curve-fittings between AI and each soil microbial metabolic index (respiration, CLI, qCO_2_, and the ratio of MBC to SOC). Linear correlation analyses (SPSS 20.0; IBM SPSS Statistics 20, Armonk, USA) were used to further explore the relationships between microbial metabolic rates and plant and soil variables. The Piecewise (segmented) Linear Regression module (R 3.02, R Core Team, 2013) was used to determine the potential AI threshold for each microbial metabolic index and other measurements (e.g., basal respiration, SIR, CLI, qCO_2_, soil C:N ratio and MBC:SOC ratio). Ordinary least squares regression analysis was conducted to examine all the linear relationships between AI and soil characteristics. Differences in slopes of ordinary least squares regressions were tested by one-way analysis of covariance.

## Results

### Changes of basal respiration and SIR with aridity

**Figure 2:**
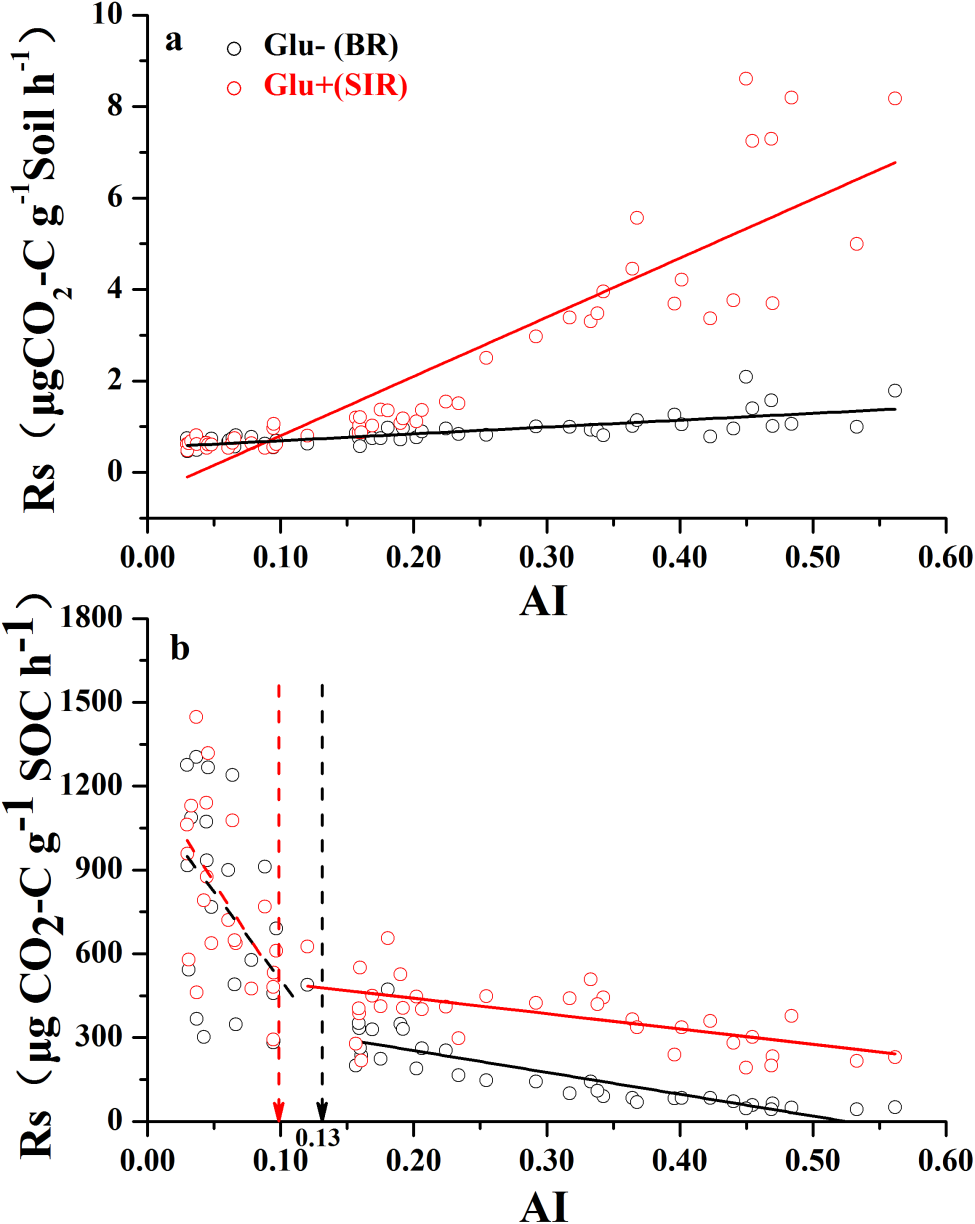
The variation of soil respiration rate (Rs) along the aridity gradient (AI) for soils from 56 locations along the sampling transect. The variation of soil respiration rates (Rs) along the aridity gradient (AI) for soils from 56 locations along the sampling transect. In (A), the soil respiration rate is expressed as the rate of CO_2_-C release per unit of soil mass. In (B), the soil respiration rate is expressed as the rate of CO_2_-C release per unit of soil organic carbon. The black dots represent basal soil respiration without glucose addition (Glu−) and the red dots represent substrate-induced respiration rate with glucose addition (Glu+). The vertical dotted dash line indicates AI threshold value determined by the break-point in segmented linear regression. The *R^2^* and *P*-values were obtained from linear regressions.

Both soil mass-based microbial basal respiration and SIR (Fig. 2A) linearly increased with increasing AI values (where a lower AI value indicates a higher level of aridity and vice versa). However, when both respiration rates were expressed as CO_2_ production rate per unit of SOC, the pattern of regression with AI reversed and became nonlinear (Fig. 2B). There were AI thresholds at AI = 0.13 and AI = 0.10 for basal respiration and SIR, respectively (Table S1). The SOC-based basal respiration was negatively and linearly correlated with AI when AI > 0.13, and was not significantly correlated with AI when AI < 0.13. Similarly, the SOC-based SIR was negatively and linearly correlated with AI when AI > 0.10, and was not significantly correlated with AI when AI < 0.10. Furthermore, microbial respiratory response to glucose addition was close to zero when AI < 0.10, but was much apparent when AI > 0.10. In other words, the relationship between AI and SOC-based respiration rates showed a nonlinear pattern with AI thresholds, which are close to the widely used AI = 0.2 as a boundary AI value between arid land and semi-arid land (United Nations Environment Programme (UNEP), 1992).

### Changes of CLI and qCO_2_ with aridity and other variables

**Figure 3:**
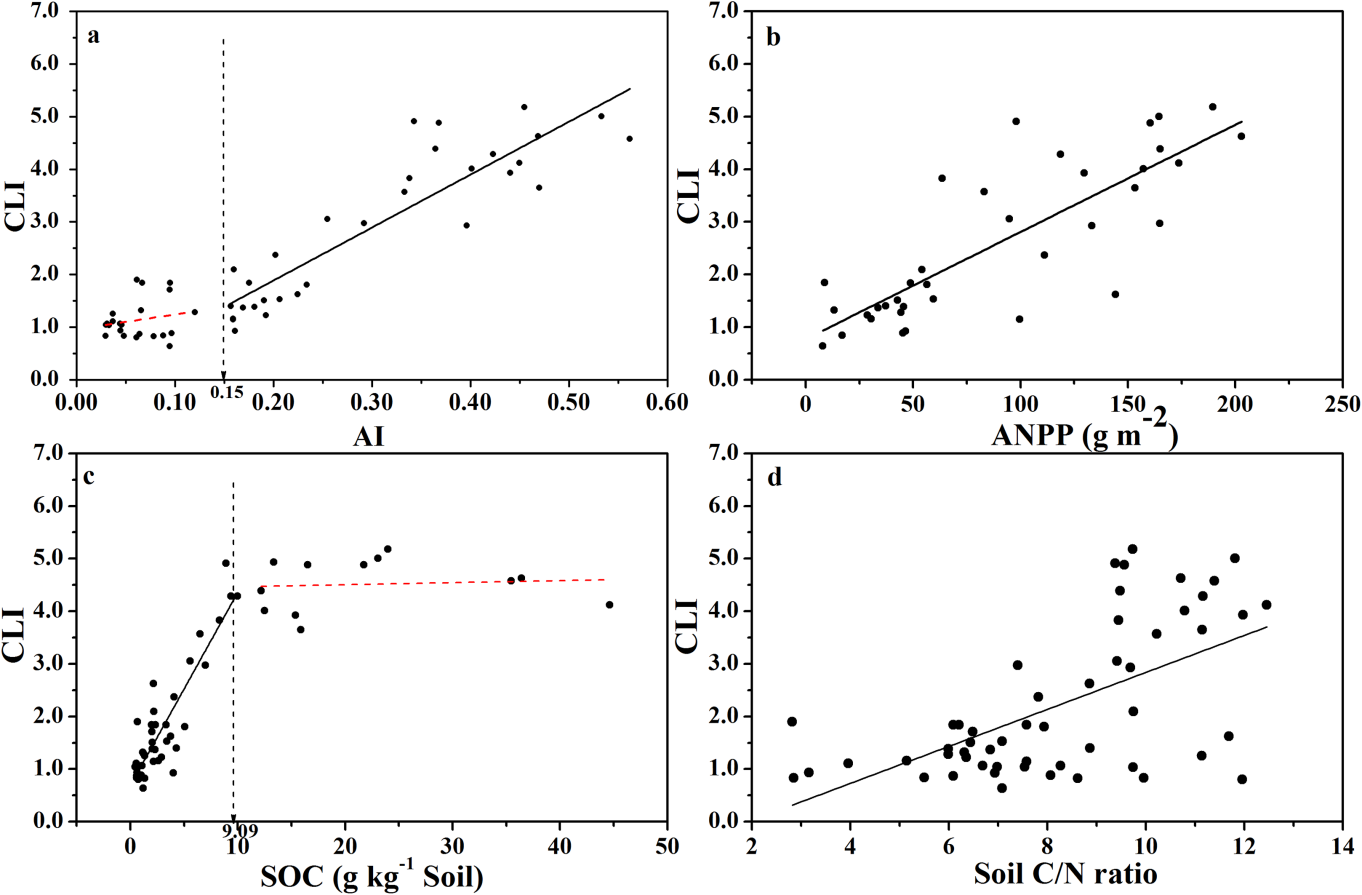
Changes of CLI with AI, ANPP, SOC, and soil C:N ratio for soils from the 56 locations along the sampling transect. Changes of the Carbon Limitation Index (CLI) with the aridity index (AI) (A), annual net primary productivity (ANPP) (B), soil organic carbon content (SOC) (C), and soil C:N ratio (D) for soils from the 56 locations along the sampling transect. The vertical dotted dash line indicates the AI threshold value determined by the break-point in segmented linear regression. The *R^2^* and *P*-values were obtained from linear regressions.

The CLI increased with increasing AI (Fig. 3A), indicating that the degree of microbial carbon limitation increased as the level of aridity decreased. More importantly, there was a threshold at AI = 0.15 (Table S1), showing that CLI increased linearly with AI when AI > 0.15, while CLI remained low when AI < 0.15. In addition, CLI showed a positive linear correlation with ANPP (Fig. 3B), indicating that soil microbial respiration became more carbon-limited in ecosystems with higher primary production or more plant C input. Besides, CLI increased linearly as SOC content increased up to 9.0 g C kg^−1^ of soil (Fig. 3C; Table S1), and CLI tended to level off above 9.0 g C kg^−1^ of soil, indicating a threshold at 9.0 g C kg^−1^ soil in the relationship between CLI and SOC content. There was a weaker positive linear relationship between CLI and soil C:N ratio (Fig. 3D).

**Figure 4:**
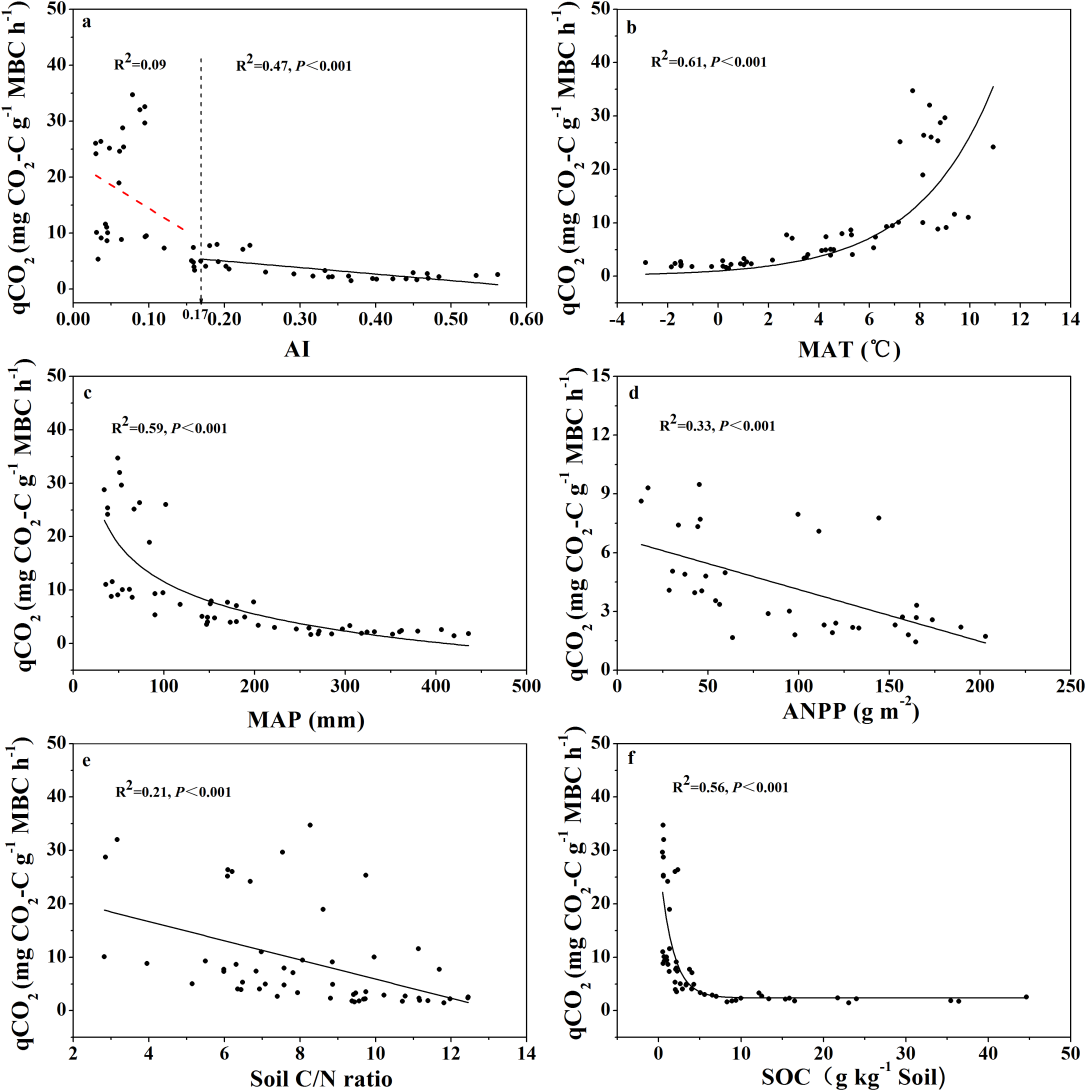
Variations and correlations of qCO_2_ with AI, MAT, MAP, ANPP, soil C:N ratio, and SOC for soils from the 56 locations along the sampling transect. Variations and correlations of microbial metabolic quotient (qCO_2_) with aridity index (AI) (A), mean annual temperature (MAT) (B), mean annual precipitation (MAP) (C), annual net primary productivity (ANPP) (D), soil C:N ratio (E), and soil organic carbon content (SOC) (F) for soils from the 56 locations along the sampling transect. The vertical dotted dash line indicates AI threshold value determined by the break-point in segmented linear regression. The *R^2^* and *P*-values were obtained from either linear regressions or curve-linear regressions.

The relationship between qCO_2_ and AI again indicated a threshold at AI = 0.17 (Fig. 4; Table S1), where qCO_2_ decreased linearly as AI increased above the threshold, while it decreased sharply as AI increased below the threshold of 0.17 (although in the latter case the relationship was not statistically significant) (Fig. 4A). Because AI is negatively related to mean annual temperature and positively related to MAP (Fig. S2), qCO_2_ was positively correlated with mean annual temperature and negatively correlated with MAP (Figs. 4B and 4C). Furthermore, qCO_2_ showed a negative linear correlation with ANPP (Fig. 4D) and soil C:N ratio (Fig. 4E), but a non-linear inverse relationship with SOC content (Fig. 4F) (high qCO_2_ values were associated with low SOC contents and vice versa).

### Changes of microbial biomass with aridity and other variables

Both ANPP and soil mass-based MBC increased linearly with increasing AI values (Figs. S3A and S3B), indicating that climate aridity had important control over ANPP and soil MBC. Similarly, SOC and SON content both increased curve-linearly with increasing AI values (Figs. S3C and S3D), suggesting that higher ANPP at higher AI values (higher water availability) would support higher soil MBC and maintain higher SOC and SON contents. However, the relationship between soil C/N ratio with AI showed a clear threshold at AI = 0.17 (Fig. S3E; Table S1). The soil C/N ratio increased linearly with increasing AI values when AI > 0.17, while there was no clear relationship when AI < 0.17.

**Figure 5:**
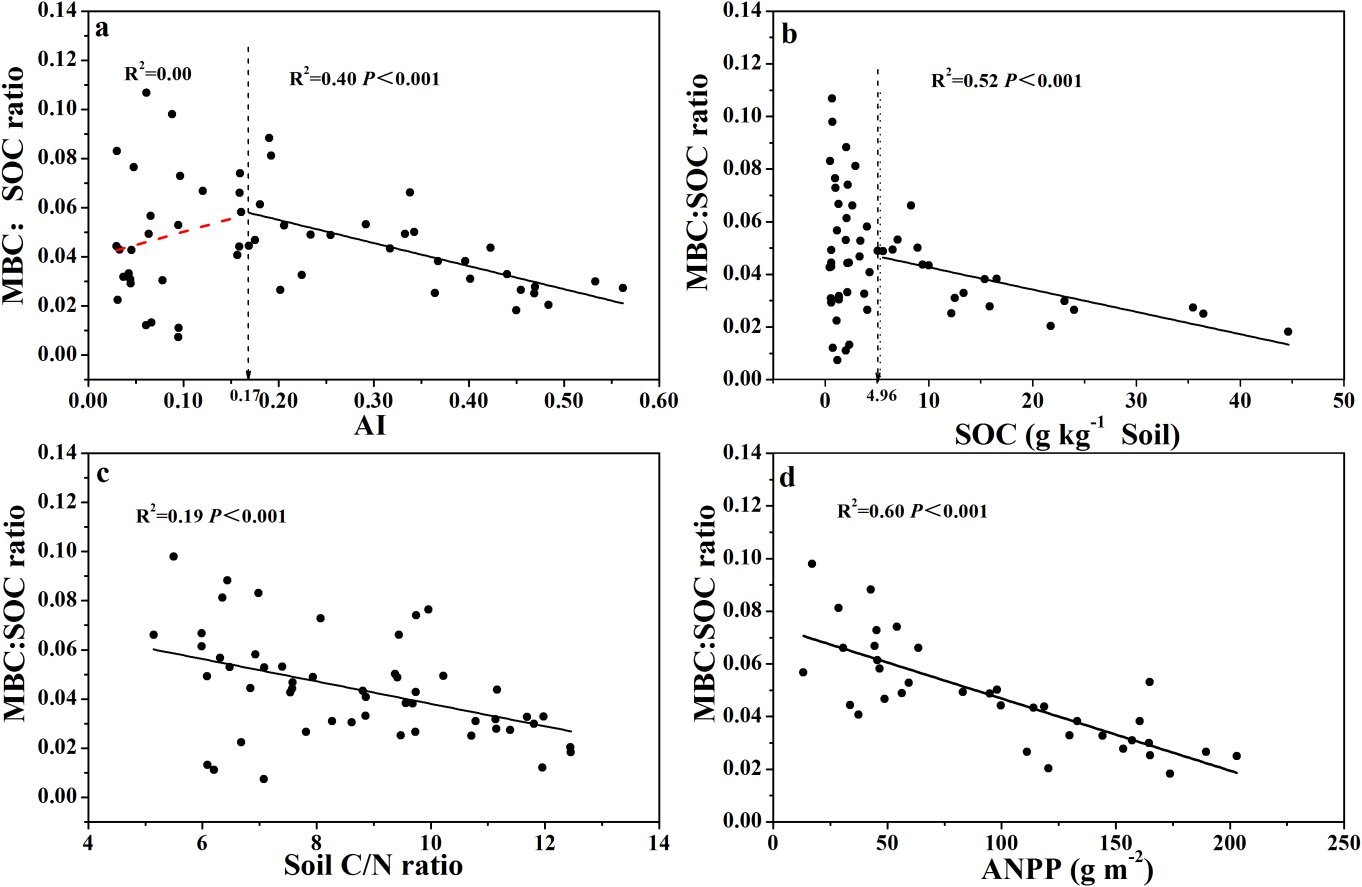
Variation and correlation patterns of the MBC:SOC ratio with AI, SOC, soil C:N ratio, and ANPP for soils from the 56 locations along the sampling transect. Variation and correlation patterns of the MBC:SOC ratio with aridity index (AI) (A), soil organic carbon content (SOC) (B), soil C:N ratio (C), and annual net primary productivity (ANPP excluding zero values) (D) for soils from the 56 locations along the sampling transect. The vertical dotted dash line indicates AI threshold value determined by the break-point in segmented linear regression. The *R^2^* and *P*-values were obtained from linear regressions.

Similarly, the relationship between MBC:SOC ratio with AI showed a clear threshold at AI = 0.17 (Fig. 5A; Table S1), where the MBC:SOC ratio decreased linearly with increased AI above the threshold; but tended to increase with increased AI below the threshold. Similarly, the MBC:SOC ratio decreased linearly with increased SOC content at SOC contents higher than 5.0 g C kg^−1^soil, but with no clear relationship at SOC contents lower than 5.0 g C kg^−1^ soil (Fig. 5B; Table S1), showing a likely threshold of SOC content at 5.0 g C kg^−1^soil. The MBC:SOC ratio also showed a linearly decreasing trend with increasing soil C:N ratios (*R*^2^= 0.19, *P* < 0.001) (Fig. 5C) and ANPP (*R*^2^= 0.60, *P* < 0.001) (Fig. 5D).

## Discussion

### Soil basal respiration and substrate-induced respiration along the aridity gradient

Soil microbial respiration is a central process in terrestrial ecosystems, which is closely connected to virtually all biogeochemical cycles. Here, we made an attempt to address the research question: how does soil microbial respiration change across a wide aridity gradient? We found that soil mass-based respiration rates (both basal and SIR) increased as AI values increased (Fig. 2A), largely because locations with higher AI values had higher ANPP and SOC content, both of which were highly positively related to soil mass-based respiration rates (Fig. S3). It should be noted that the AI is the ratio of annual precipitation to annual PET, a lower AI value indicates a higher level of aridity. It is widely known that soil microbial respiration rates are primarily driven by SOC contents, which are supported by primary production (Raich & Schlesinger, 1992; Colman & Schimel, 2013).

However, when we expressed basal and SIR rates as per unit of SOC, different patterns emerged (Fig. 2B). SOC-specific basal and SIR rates decreased as AI value increased, and each showed a threshold at AI = 0.10 and 0.13, respectively (Fig. 2B; Table S1). The SOC-based basal respiration and SIR were negatively and linearly correlated with AI above the thresholds, and were not significantly related with AI below the thresholds. It was also clear that the microbial respiratory response to glucose addition was very weak when AI < 0.10, but was much stronger when AI > 0.10. Given that the SOC-specific basal respiration rate is a surrogate for SOC decomposition rate that we measured under standardized temperature and moisture conditions (temperature at 25 °C and moisture at 60% WHC), this pattern indicates that this standardized microbial decomposition rate of SOC decreases as aridity decreases (or as AI increases) when AI > 0.13. However, this trend disappeared when AI < 0.13. Although the exact mechanisms behind this pattern are unknown at this point, two possible explanations can be offered here. First, microbes living under drought-dominant conditions (AI < 0.1) are expected to have higher respiration rates and lower carbon use efficiencies due to drought-tolerance (Schimel, Balser & Wallenstein, 2007), because all three known microbial physiological features related to drought-tolerance involve releases of extra amount of energy and nutrients (Harris, 1981; Schimel, Balser & Wallenstein, 2007). Therefore, microbial drought-tolerance can be one of the mechanisms behind this pattern. Second, this SOC-specific respiration pattern along the AI gradient can result from differences in microbial community composition driven by the aridity gradient (Goberna et al., 2014; Yao et al., 2017; Ochoa-Hueso et al., 2018). This possibility is indirectly supported by the results presented in Fig. 5B which showed that, when SOC content drops below 5.0 g kg^−1^ of soil (corresponds to AI value of 0.2), SOC is no longer the main substrate basis for microbes: they live off of something else (e.g., chemo-autotrophs and/or photo-autotrophs), which is supported by a recent finding that some microbes in extremely try desert have the capability of using atmospheric trace gases as their energy source (Bay, Ferrari & Greening, 2018). This point is further enforced by the recent finding that desert microbial communities clearly have much lower network complexity than semi-arid grasslands (Wang et al., 2017). Clearly, a better mechanistic understanding about the SOC-specific respiration pattern along the AI gradient warrants more studies.

### Microbial metabolic quotient and carbon limitation index along the aridity gradient

Soil microbial metabolic quotient (qCO_2_) has become an important index in soil microbiology and biogeochemistry since being introduced by Anderson & Domsch (1985). Recently, qCO_2_ has been used as a crucial parameter in models of soil carbon cycling (Allison, Wallenstein & Bradford, 2010; Wang, Post & Mayes, 2013; Wieder, Bonan & Allison, 2013; Sulman et al., 2014), largely because qCO_2_ is a surrogate for both microbial enzyme production rate and microbial biomass turnover rate, and both rates are difficult to directly quantify. In this current study we aimed to address the question: what was the pattern of change in qCO_2_ across the wide aridity gradient? We found that qCO_2_ was positively related with mean annual temperature and negatively correlated with MAP (Figs. 4B and 4C), which is in accordance with published reports (Insam, 1990; Xu et al., 2017). More importantly, we found that qCO_2_ decreased linearly as AI increased when AI > 0.17, and that qCO_2_ tended to decrease sharply as AI increased when AI < 0.17 (but the correlation was not statistically significant) (Fig. 4A). This pattern again indicated a threshold at AI = 0.17. This pattern of qCO_2_ change along the aridity gradient is quite similar to the pattern with SOC-specific microbial respiration. Logically, the two possible explanations behind this pattern given in the previous paragraph should also apply to qCO_2_ here; that is, microbial tolerance to drought may result in higher qCO_2_ values when AI < 0.17, and the aridity gradient may have resulted in different microbial community composition (Yao et al., 2017; Ochoa-Hueso et al., 2018) that sharply differ in metabolic rates.

We found that the CLI increased as AI increased (Fig. 3A), indicating that the degree of microbial carbon limitation increased as the level of climate aridity decreased. We also found an AI threshold at AI = 0.15 for CLI. CLI increased linearly with AI when AI > 0.15, and CLI remained low when AI < 0.15, indicating that soil microbial respiration was not limited by available carbon substrates in arid regions. Furthermore, CLI showed a positive linear correlation with ANPP (Fig. 3B). This indicates an apparent paradox in that soil microbial respiration becomes more carbon-limited in ecosystems with increasingly higher primary production or higher substrate supply. This raises more questions. Why do soil microbes display a higher level of carbon starvation at locations with higher substrate input? Why do microbes at the very dry end of the gradient (AI < 0.15) show little carbon limitation where primary production is very low? Is it because all drought-tolerant microbes are oligotrophs which have much longer lag-time to respond to substrate changes, and copiotrophs (which often show fast responses to substrate addition) become more abundant as water availability improves (Hattori, 1984)? Is it because the stronger competition between soil microbes and plants for mineral nutrients when AI > 0.15 differentially selects for more carbon-starved microbes as predicted in a theoretical analysis, which postulated that soil microbes must be carbon-limited and plants much be nutrient-limited if both persist (Daufresne & Loreau, 2001)? Our data (Fig. 5A) indeed implied that, when SOC content drops below 5.0 g kg^−1^ soil (corresponds to AI < 0.15), SOC is no longer the main substrate basis for soil microbes. In other words, microbes at the very dry end may consist of chemo-autotrophs and/or photo-autotrophs which do not use SOC as their substrates (Bay, Ferrari & Greening, 2018), but indeed, may form carbon substrates for heterotrophic microbes thereby alleviating their carbon limitation. Ultimately, finding answers to these questions requires more investigations.

### Microbial biomass carbon along the aridity gradient

One of our objectives in this study was to explore the relationship between soil microbial biomass and climate aridity. We found that soil mass-based MBC increased linearly with increasing AI values, and similarly, ANPP and SOC content also increased as AI values increased (Figs. S3A–S3C). Climate aridity exerts a major control on soil mass-based MBC, ANPP, and SOC content, suggesting that higher ANPP at higher AI values (higher water availability) supports higher soil MBC and maintains higher SOC content. These trends are in agreements with published results (Colman & Schimel, 2013; Xu et al., 2014).

The MBC:SOC ratio, however, showed a non-linear relationship with AI (Fig. 5A): the MBC:SOC ratio decreased linearly as AI increased when AI > 0.17, but, increased as AI increased when AI < 0.17. Again, an AI threshold value was observed at 0.17 for the relationship with MBC:SOC ratio. The trend shown at the right side of the AI threshold (AI > 0.17, Fig. 5A) is in accordance with the result of Insam, Parkinson & Domsch (1989), who also showed lower MBC:SOC ratios in ecosystems with relatively higher AI values. Intriguingly, the trend shown at the left side of the AI threshold (AI < 0.17, Fig. 5A) is new, as to the best of our knowledge, and requires more discussion. Why does the MBC:SOC ratio scatter widely and tend to decrease as AI decreases, when AI < 0.17 (the opposite trend to AI > 0.17)? According to the simulation (modeling) results of Xu et al. (2014), the MBC:SOC ratio is primarily regulated by two variables: (1) the quality of soil organic substrates (approximated with C:N ratio), and (2) cumulative microbial activity index in days per year (primarily related to water and temperature status). With their model, Xu et al. (2014) were able to accurately predict MBC:SOC ratios at the biome scale, with higher ratios in ecosystems with lower soil C:N ratios and a lower (or shorter) cumulative microbial activity index. In the case of our current study, their model would predict higher MBC:SOC ratios as AI further decreases from 0.17, because lower AI values would directly translate to smaller (shorter) cumulative microbial activity index and also correspond to lower soil C:N ratios or higher substrate quality (AI values were positively correlated with C:N ratio, *R*^2^= 0.81, Fig. S2E). Apparently, the model prediction does not fit with our empirical data when AI < 0.17, even though there were a few data points of very high MBC:SOC ratios (Fig. 5A). The model would not predict the scatter, nor the declining trend as AI further decreases from 0.17. Furthermore, if chemo-autotrophs and/or photo-autotrophs are an important component of the microbial community in soils with an AI < 0.17, this would also result in relatively high MBC:SOC ratios, higher than predicted based on the regression shown for AI > 0.17. It is clear that more studies are required to affirm the real mechanisms driving this pattern.

## Conclusions

We identified aridity thresholds for each of the three soil microbial metabolic indices (SOC-based respiration, qCO_2_, and the MBC:SOC ratio) along the aridity gradient. These metabolic indices linearly declined when AI values were above the corresponding thresholds, but did not show any consistent patterns when AI values were smaller than the threshold of 0.1. We also found an apparent paradox: soil microbes showed a higher level of carbon limitation at locations with higher substrate supply and relatively lower level of water limitation when AI was above the corresponding threshold, that is, soil microbes become more starved when they live in ecosystems with more substrates for them. The mechanistic causes of this paradox warrant further studies. Nevertheless, the increasing level of carbon limitation did correspond to the declining trend of the three metabolic indexes along the AI gradient, which indicates that the carbon limitation may exert a major control on microbial metabolisms. Overall, microbial metabolisms under extremely dry condition were fundamentally different than under semi-arid conditions.

## Supplemental Information

10.7717/peerj.6712/supp-1 Supplemental Information 1 Raw data of a sampling transect of 3,200 km across arid and semi-arid regions in Northern China.

10.7717/peerj.6712/supp-2 Supplemental Information 2 Raw data of the variation of soil respiration rate (Rs) along the aridity gradient (AI) for soils from 56 locations along the sampling transect. The soil respiration rate was expressed as the rate of CO_2_-C release per unit of soil mass and the rate of CO_2_-C release per unit of soil organic carbon, respectively. Basal soil respiration without glucose addition was named as Glu− and the substrate-induced respiration rate with glucose addition was named as Glu+.

10.7717/peerj.6712/supp-3 Supplemental Information 3 Raw data of changes of CLI with AI, ANPP, and soil C:N ratio for soils from the 56 locations along the sampling transect. Changes of carbon limitation index (CLI) with aridity index (AI), annual net primary productivity (ANPP), soil organic carbon content (SOC), and soil C:N ratio for soils from the 56 locations along the sampling transect.

10.7717/peerj.6712/supp-4 Supplemental Information 4 Raw data of variations and correlations of qCO_2_ with AI, MAT, MAP, ANPP. Variations and correlations of microbial metabolic quotient (qCO_2_) with aridity index (AI), mean annual temperature (MAT), Mean annual precipitation (MAP), annual net primary productivity (ANPP), soil C:N ratio, and soil organic carbon content (SOC) for soils from the 56 locations along the sampling transect.

10.7717/peerj.6712/supp-5 Supplemental Information 5 Raw data of variations of ANPP, MBC, and SOC along the aridity gradient AI for soils from the 56 locations along the sampling. Variations of annual net primary productivity (ANPP), microbial biomass carbon (MBC) , and soil organic carbon content (SOC) along the aridity gradient (AI) for soils from the 56 locations along the sampling transect.

10.7717/peerj.6712/supp-6 Supplemental Information 6 Raw data of variation and correlation patterns of the MBC:SOC ratio with AI, SOC, soil C:N ratio, and ANPP for soils from the 56 locations along the sampling transect. Variation and correlation patterns of the MBC:SOC ratio with aridity index (AI), soil organic carbon content (SOC), soil C:N ratio, and annual net primary productivity (ANPP) for soils from the 56 locations along the sampling transect.

10.7717/peerj.6712/supp-7 Supplemental Information 7 Variation and correlation of MAT with MAP. Figure S1. Variation and correlation of mean annual temperature (MAT) with mean annual precipitation (MAP) for soils from the 56 locations along the sampling transect. The *R^2^* and *P*-values were obtained from linear regression.

10.7717/peerj.6712/supp-8 Supplemental Information 8 Variations of MAP and MAT along AI in the transect. Figure S2. Variations of mean annual precipitation (MAP) (Fig. S2A) and mean annual temperature (MAT) (Fig. 2B) along the aridity gradient (AI) for soils from the 56 locations along the sampling transect. The *R^2^* and *P*-values were obtained from either linear regressions or curve-linear regressions.

10.7717/peerj.6712/supp-9 Supplemental Information 9 Variations of ANPP, MBC, SOC, SON, and soil C:N ratio along the AI for soils from the 56 locations along the sampling transect. Figure S3. Variations of annual net primary productivity (ANPP, locations with zero ANPP values were excluded) (Fig. S3A), microbial biomass carbon (MBC) (Fig. S3B), soil organic carbon content (SOC) (Fig. S3C), soil total nitrogen content (Fig. S3D), and soil C:N ratio (Fig. S3E) along the aridity gradient (AI) for soils from the 56 locations along the sampling transect. The vertical dotted dash line indicates AI threshold value determined by the break-point in segmented linear regression. The *R^2^* and *P*-values were obtained from either linear regressions or curve-linear regressions.

10.7717/peerj.6712/supp-10 Supplemental Information 10 Results of Segmented (Piecewise) Linear Regressions for determining AI threshold (THR) values to soil microbial metabolic indices. Table S1. Results of Segmented (Piecewise) Linear Regressions for determining AI threshold (THR) values to soil microbial metabolic indices. The degree of freedom (d*f*) = 55 and *P* < 0.001 for all regressions. AI, aridity index; BR, basal respiration; SIR, substrate-induced respiration; CLI, carbon limitation index; qCO_2_, soil microbial metabolic quotient; MBC, soil microbial biomass carbon; SOC, soil organic carbon.

## Additional Information and Declarations

### Data Availability

The following information was supplied regarding data availability: The raw data is available in the Supplemental Materials.
