# Supplementary figures and images for: Aridity thresholds of soil microbial metabolic indices along a 3,200 km transect across arid and semi-arid regions in Northern China
Source: PeerJ. 2019 Apr 9;7:e6712. doi: 10.7717/peerj.6712 (PMC6461032; doi:10.7717/peerj.6712)

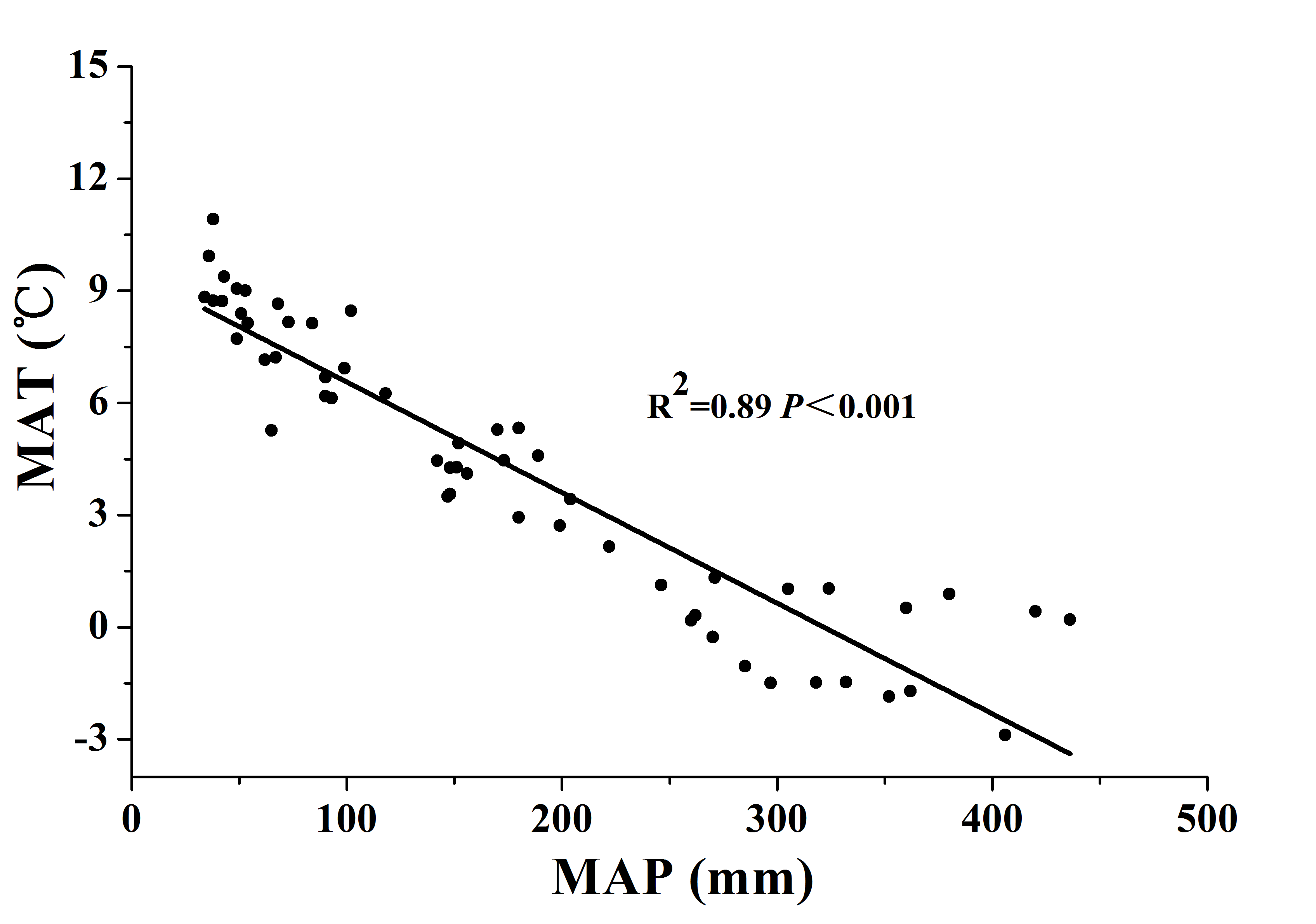

Supplement: Supplemental Information 7 — Figure S1. Variation and correlation of mean annual temperature (MAT) with mean annual precipitation (MAP) for soils from the 56 locations along the sampling transect. The R2 and P-values were obtained from linear regression. [file peerj-07-6712-s007.png]

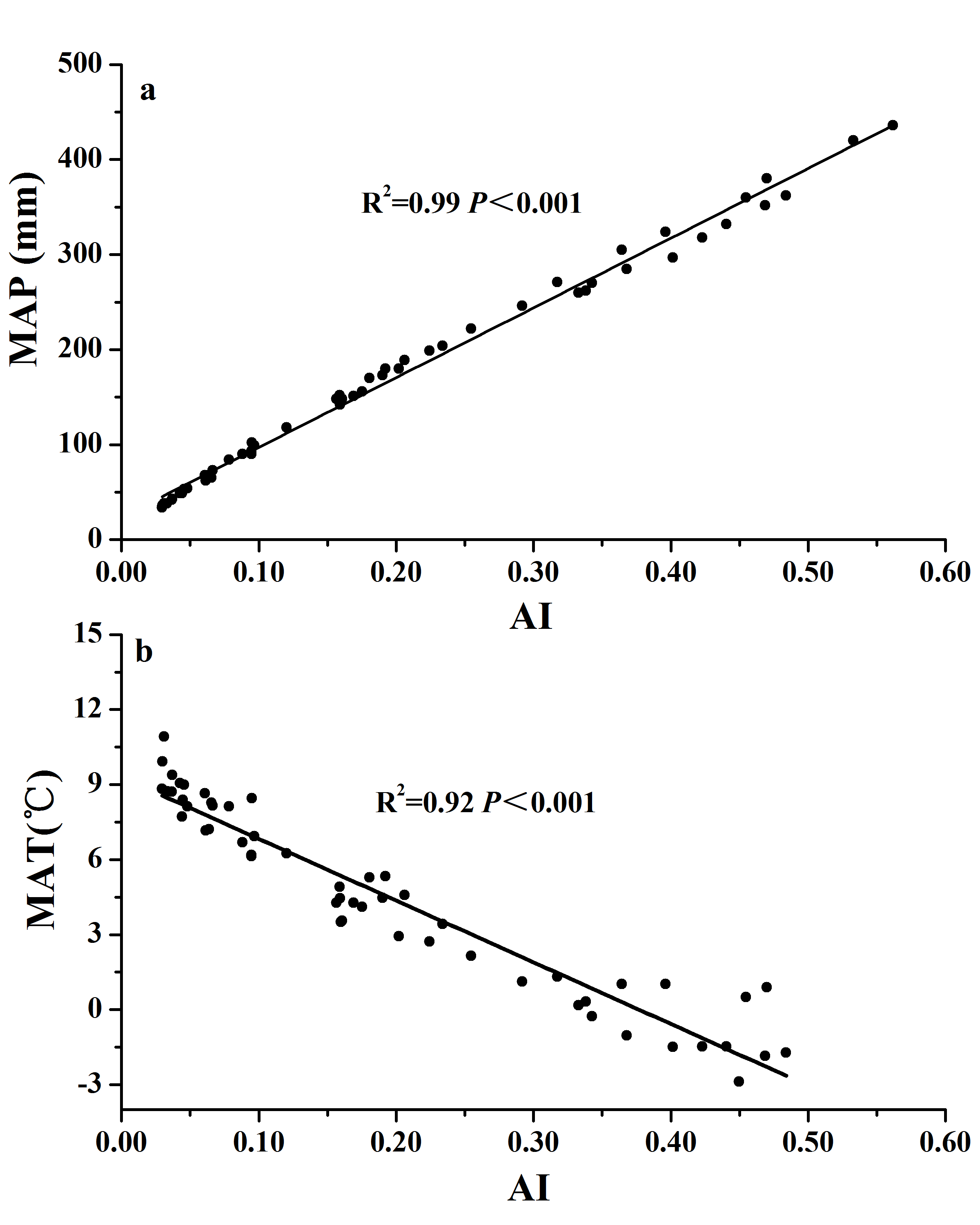

Supplement: Supplemental Information 8 — Figure S2. Variations of mean annual precipitation (MAP) (Fig. S2A) and mean annual temperature (MAT) (Fig. 2B) along the aridity gradient (AI) for soils from the 56 locations along the sampling transect. The R2 and P-values were obtained from either linear regressions or curve-linear regressions. [file peerj-07-6712-s008.png]

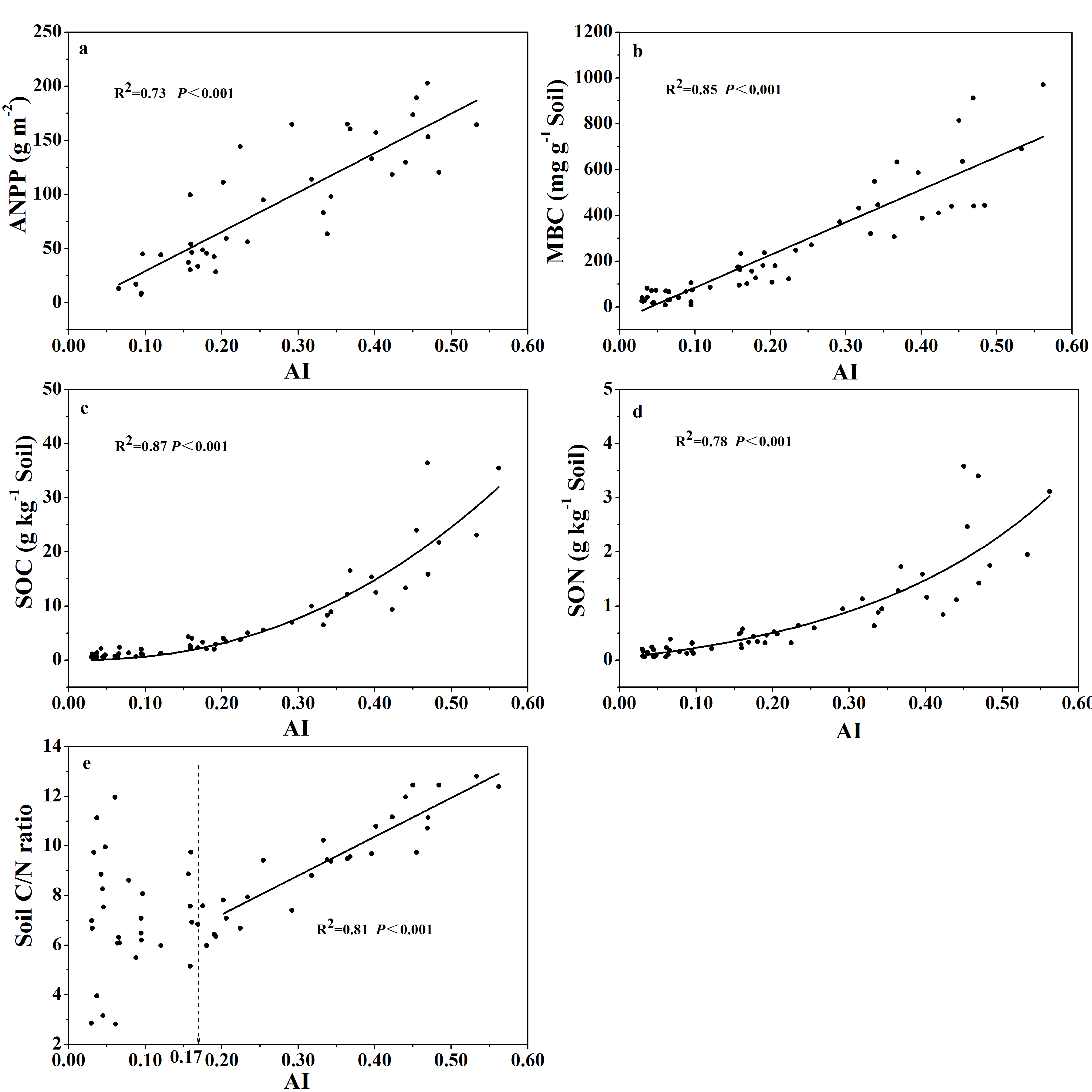

Supplement: Supplemental Information 9 — Figure S3. Variations of annual net primary productivity (ANPP, locations with zero ANPP values were excluded) (Fig. S3A), microbial biomass carbon (MBC) (Fig. S3B), soil organic carbon content (SOC) (Fig. S3C), soil total nitrogen content (Fig. S3D), and soil C:N ratio (Fig. S3E) along the aridity gradient (AI) for soils from the 56 locations along the sampling transect. The vertical dotted dash line indicates AI threshold value determined by the break-point in segmented linear regression. The R2 and P-values were obtained from either linear regressions or curve-linear regressions. [file peerj-07-6712-s009.png]
