# Supplementary material for: Aridity thresholds of soil microbial metabolic indices along a 3,200 km transect across arid and semi-arid regions in Northern China
Source: PeerJ. 2019 Apr 9;7:e6712. doi: 10.7717/peerj.6712 (PMC6461032; doi:10.7717/peerj.6712)
Supplement: Supplemental Information 10 — Table S1. Results of Segmented (Piecewise) Linear Regressions for determining AI threshold (THR) values to soil microbial metabolic indices. The degree of freedom (df) = 55 and P < 0.001 for all regressions. AI, aridity index; BR, basal respiration; SIR, substrate-induced respiration; CLI, carbon limitation index; qCO2, soil microbial metabolic quotient; MBC, soil microbial biomass carbon; SOC, soil organic carbon. [file peerj-07-6712-s010.docx]

**Supplemental Information**

**File-4:**

**Table S1.** Results of Segmented (Piecewise) Linear Regressions for determining AI threshold (THR) values to soil microbial metabolic indices. The degree of freedom (df) =55 and P<0.001 for all regressions. AI: aridity index; BR: basal respiration; SIR: substrate-induced respiration; CLI: carbon limitation index; qCO_2_: soil microbial metabolic quotient; MBC: soil microbial biomass carbon; SOC: soil organic carbon.

|  | **Break point** | **Value<THR** | **Value>THR** |
| --- | --- | --- | --- |
|  | **THR** | **Equation** | **Equation** |
| AI-BR | 0.13±0.03 | y=1164.60-6883.50x,  R^2^=0.17 | y=432.57-838.23x,  R^2^=0.73 |
| AI-SIR | 0.10±0.01 | y=1170.79-6305.18x,  R^2^=0.28 | y=594.40-661.64x,  R^2^=0.57 |
| AI-CLI | 0.15±0.06 | y=0.96+2.80x,  R^2^=0.00 | y=-0.12+10.05x,  R^2^=0.80 |
| AI-qCO_2_ | 0.17±0.03 | y=22.76-83.46x,  R^2^=0.09 | y=7.33-11.66x,  R^2^=0.47 |
| AI-C:N ratio | 0.17±0.03 | NA | y=4.10-15.67x,  R^2^=0.81 |
| AI-MBC:SOC ratio | 0.17±0.06 | y=0.040-0.11x,  R^2^=0.00 | y=0.07-0.09x,  R^2^=0.40 |
| SOC-CLI | 9.09±0.97 | y=0.69-0.37x,  R^2^=0.81 | y=4.314 |
| SOC-MBC:SOC ratio | 4.96±0.48 | NA | y=0.05-8.23×10^-4^x,  R^2^=0.52 |
